# Artificial Intelligence for Antimicrobial Resistance Prediction: Challenges and Opportunities towards Practical Implementation

**DOI:** 10.3390/antibiotics12030523

**Published:** 2023-03-06

**Authors:** Tabish Ali, Sarfaraz Ahmed, Muhammad Aslam

**Affiliations:** 1 Department of Civil & Environmental Engineering, Hanyang University, Seoul 04763, Republic of Korea; 2 Department of Electronics & Computer Engineering, Hanyang University, Seoul 04763, Republic of Korea; 3 Department of Artificial Intelligence, Sejong University, Seoul 05006, Republic of Korea

**Keywords:** antimicrobial resistance genes, artificial intelligence, deep learning, machine learning, challenges and opportunities

## Abstract

Antimicrobial resistance (AMR) is emerging as a potential threat to many lives worldwide. It is very important to understand and apply effective strategies to counter the impact of AMR and its mutation from a medical treatment point of view. The intersection of artificial intelligence (AI), especially deep learning/machine learning, has led to a new direction in antimicrobial identification. Furthermore, presently, the availability of huge amounts of data from multiple sources has made it more effective to use these artificial intelligence techniques to identify interesting insights into AMR genes such as new genes, mutations, drug identification, conditions favorable to spread, and so on. Therefore, this paper presents a review of state-of-the-art challenges and opportunities. These include interesting input features posing challenges in use, state-of-the-art deep-learning/machine-learning models for robustness and high accuracy, challenges, and prospects to apply these techniques for practical purposes. The paper concludes with the encouragement to apply AI to the AMR sector with the intention of practical diagnosis and treatment, since presently most studies are at early stages with minimal application in the practice of diagnosis and treatment of disease.

## 1. Introduction

The emergence and spread of antimicrobial resistance (AMR) is a major public health challenge presently faced by the world. AMR originates when micro-organisms, such as bacteria, viruses, fungi, and parasites, become resistant to the drugs that were once effective in treating infections caused by these micro-organisms. Globally, 1.27 million deaths have occurred each year due to AMR [1]. It is also important to note that the challenge of antimicrobial resistance is limited not only to bacteria but also to viruses. Recently, emerging and re-emerging viruses such as the COVID-19 pandemic, caused by SARS-CoV-2, have shown the importance of continuous research and development in developing new antiviral agents and vaccines to fight against such events. These events have forced the authorities to take necessary actions to tackle these challenges and propose appropriate diagnostic methods [2,3,4,5]. These approaches will make it possible to take the right steps to minimize the threat of AMR, using precautions and appropriate antibiotics. 

However, antibiotic resistant genes (ARG) identification poses challenges as it requires high accuracy for practical treatment and robustness to quickly identify the problem [6]. Currently, characterization and diagnostic techniques used in laboratories do not provide enough information to effectively perform surveillance [6]. Furthermore, tests generate inconsistent results depending on environment and laboratory setup [7,8]. Data obtained from high sequencing such as whole-genome sequence, along with laboratory techniques, can be used to investigate the genetic variants of widespread AMR [6]. 

Recently, with the advancement in technology, computing power and data storage of computers have been immensely increased, allowing tremendous amounts of data to be processed and analyzed in a little amount of time. Therefore, the applications of artificial intelligence (AI) techniques, especially machine-learning (ML) and deep-learning (DL) techniques, are being used across different fields [9,10,11]. The application of AI is also increasing in the medical sector [12,13,14]. The metaverse is being developed for intelligent healthcare [13]. The authors in [12] listed Food and Drug Administration (FDA)-approved AI/ML-enabled devices across different medical fields. Significant increase in the approval of such AI-enabled devices is observed from 2018 onwards which constitutes about 85% of all approved devices. [12]. In total, around 531 AI/ML-enabled devices have been approved, and the majority of the approved devices are related to radiology. Among the others listed, five AI/ML-enabled approved devices are related to microbiology and four are related to pathology [12]. 

The ML/DL models extract interesting underlying relationships and patterns between features and prospective outcomes. There are three types of deep-learning models [9,10,11]: Supervised learning models are trained using input features with a corresponding target output to approximate and find underlying non-linear relationship, and these types of models are useful in regression and prediction. Unsupervised learning is trained only on input features to make clusters or groups among the input features. Reinforcement learning is trained based on rewards and penalties, mostly suitable for control and operations. 

There are different ways in which machine learning has been applied to AMR. For example, AMR is being studied by sequence-based application of AI in [15,16,17]. AI has been applied to design new antibiotics, and generates a synergy of a combination of drugs [18,19]. The machine-learning algorithms analyze patterns in data on antimicrobial use and resistance to predict which micro-organisms are likely to develop resistance to certain drugs. This can help healthcare providers and policymakers make informed decisions about which drugs to use and how to use them [6]. Machine-learning models are used for surveillance of antimicrobial resistance [6]. These models analyze large amounts of data on antimicrobial use and resistance to identify emerging resistance patterns and potential hotspots of AMR. This helps public health authorities respond to outbreaks of resistant infections more quickly and effectively. For antimicrobial stewardship, machine learning is also used to optimize antimicrobial use in healthcare settings, for example by identifying the optimal combination of drugs to use for a particular infection or by predicting which patients are at risk of developing a resistant infection [20]. This can help reduce the overall burden of AMR.

Having mentioned these applications, the ML/DL models also encounter certain challenges during application in the field of antimicrobials [20,21]. An example of a major challenge is the availability of high-quality data on antimicrobials with balanced labels of susceptibility and resistance as well as those of intermediate category labels that overlap between susceptible and resistance [22]. Furthermore, there is the need to validate the results of machine-learning models, as the accuracy of these models can vary depending on the data and a particular algorithm applied. Additionally, the accuracies vary depending on experimental environmental and geographical locations, from where samples have been taken [21]. Improving accuracies of the models is another concern, as models with low accuracy cannot be applied for practical diagnostics/surveillance [21]. Furthermore, most of the research is limited to laboratory and experiments, and it is essential to apply these concepts to real-world problems [6,21]. Therefore, necessary research is needed to address these concerns, bridge gaps, and open new doors in the field of antibiotics.

This review article is organized as follows. Section 2 consists of details about ML/DL techniques for AMR. Section 3 will explain challenges, opportunities, and prospects. Finally, a conclusion is given in Section 4.

## 2. Artificial Intelligence (DL/ML) for Antimicrobial Resistance

Traditionally, to understand the mechanism of AMR, antimicrobial susceptibility testing (AST) is carried out based on phenotypic testing [22]. Phenotypes include information about the physical characteristics of micro-organisms, such as its shape, size and color. However, it takes considerable time to carry out this type of testing [23]. For instance, this testing takes 2 days for some bacterial pathogens, and a few weeks for slow-growing microbials [24,25]. Another type of data to study AMR is Genome sequences. Genome sequences are easier to extract owing to the reduction in costs and improved technology [26,27]. Further studies have also used environmental data such as temperature, humidity etc. to predict the occurrence of AMR [28]. 

**Figure 1:**
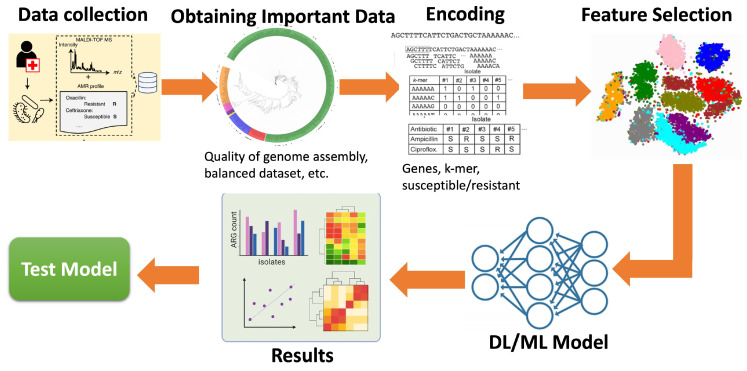
Overall process of applying machine-learning/deep-learning models in AMR identification.

AMR occurrences are predicted by different methods using these genotype data. DL/ML models are state-of-the-art tools that predict and interpret AMR [28]. These models map input features to the target labels in non-linear relationships [29]. The objective is to do regression or classification, or in some cases interpretation of the outcomes [28,30]. These models have shown good accuracy for antimicrobial susceptibility testing, if provided with enough data. Figure 1 shows the complete overview of the applications of these techniques in antimicrobial research. The following subsections give detail regarding the overall methodology of applying ML/DL for AMR prediction.

### 2.1. Overall Mechanism of ML/DL Models for the Prediction/Detection of AMR

Generally, prediction and classification problems are supervised learning problems, in which models are trained with the given input features to approximate a given target, also referred to as a “label”. The first step is data collection and data pre-processing. Data mostly consists of whole-genome sequences (WGS), and single-nucleotide polymorphisms (SNPs) with corresponding phenotypes [31,32]. For instance, in [31], WGSs used different isolates of *E. coli* strains from animals and human clinal samples. These data were both privately collected and available online as a public dataset. The aim of the paper was to study antibiotics, i.e., CIP (ciprofloxacin), CTX (cefotaxime), CTZ (ceftazidime), and GEN (gentamicin). The data consists of resistant and susceptible isolates. Features can also be generated by dividing sequences into length k, famously called k-mers [32], because it might be difficult to use complete genome strains, and also using small-length k-mers can help in identifying interesting insights of small sequences responsible for resistance [22,33,34,35,36,37].

The next important step is pre-processing and feature extraction. This consists of extracting reference alleles, variant alleles, and their position [31], after which the final SNP matrix can be built [31]. The SNPs can be encoded into chaos game representation (CGR) (A, G, C, T and N), label encoding, and one-shot encoding to train the machine-learning models [31]. For instance, to do label encoding, A, G, C, T and N in the SNP matrix can be converted into 1, 2, 3, 4, and 0 [31]. K-mers are also assigned labels with respective phenotypes and perform the encoding [32]. Data pre-processing and encoding and feature extraction all can easily be implemented using different Python packages [29]. Different machine-learning and statistical tools can also be used to generate important features. For example, a convolutional neural network (CNN) [38] with machine-learning models has been used to generate interesting features to predict AMRs. 

As well as data management, different machine-learning models have been used in the literature for the prediction/classification of AMRs [31], e.g., logistic regression (LR), support vector machine (SVM), random forest (RF), and CNN. Similarly, the authors in [30,39] used a deep-learning model, which consists of layers of artificial neurons mimicking the human brain [39]. LR, RF and SVMs are implemented by the scikit-learn Python library, whereas CNN and other deep-learning architectures can be applied with TensorFlow and Python [29]. The basic idea of all these models is to generate a mathematical relationship between input features and target labels based on available data. Therefore, the selection of relevant data is very important. After training the models several times with the training data, they can draw a mapping and learn an underlying non-learning relationship [40]. 

Once the models are trained, they are tested against unseen data, also called test data, to validate their performance before being applied in a practical purpose. Different evaluation metrics, such as root mean square error (RMS), mean absolute error (MSE), accuracy, precision, recall and confusion matrix etc., can be used to evaluate the models [40,41]. Once accuracies are satisfied, then it can be applied for practical purposes. The following sections give details of each step.

### 2.2. Data Analysis and Data Management

DL/ML models require a huge amount of genotype data for AMR prediction. The genotype data usually have output labeled phenotypes. Shotgun DNA sequences from isolates are used usually as the input sequence; also, metagenomic DNA sequences can be used [39]. Furthermore, single-nucleotide variants (SNV) can also be applied as an important input feature. Antibiotic treatment-induced transcriptional responses are also used [42,43]. Varying breakpoints may be encountered when using the phenotypic data to train machine-learning models [44], e.g., different labs test drugs depending on local prescriptions. The MIC may be different from different labs depending on one- to two-fold dilutions from one laboratory to another, contributing to noise in the phenotypic output on which the model was trained [45,46]. The standard output labels are categorized into susceptible (S) and resistant (R) with very few data included in another category, intermediate (I). This implies that DL/ML models will have good accuracies when trained with data with clear MIC distributions. On the other hand, drugs with considerable mixing/overlapping between R and S can produce low accuracy. In such cases, I might be on the verge of the boundary [45,47,48]. ML models produced more than 95% accuracy on ciprofloxacin, irrespective of clinical breakpoint. The same model, when trained for azithromycin, produced lower accuracies of between 78 and 88%. The improvement of accuracies in such cases is one of the major concerns [16] because with lower accuracies it is highly risky to implement in diagnostics. The use of appropriate data features/labels, suitable robust models and optimizing the training parameters will help to achieve higher accuracy [16].

Obtaining data is a costly as well as a time-consuming process. Therefore, another important concern is to decide the quantity of data required in training. Although there is no specific rule, it depends on the quality of data and model robustness [49]. For instance, training a model to identify methicillin resistance in *S. aureus* needs examples of less than 100 to achieve accuracy of around 99% [49]. On the other hand, *Pseudomona aeruginosa* with a more variable genome might need thousands of examples to identify long resistances [50]. Another challenging concern in terms of data is to obtain evenly distributed susceptible and resistant categories, which includes a considerable range of MICs [51]. Training models on a balanced dataset should have higher sensitivity and lower specificity, and vice versa for models trained with skewed data [43] that contain higher representation of one type of data. To achieve generalization, the diversity of isolates must also need to be understood, i.e., the mechanism of resistance that varies regionally, and avoiding training on phylogenetic confounders [45,51]. Although various techniques have been proposed recently to control the population structure, deeper research is still needed. To consider all the major *M. tuberculosis* lineages—more than 10,000 *M. tuberculosis* isolates collected by the CRyPTIC Consortium worldwide—this will obtain enough samples of pyrazinamide-resistant isolates [45,52]. 

### 2.3. Prediction Strategies

Commonly, DL/ML models are trained on constant-length continuous or binary vectors. The raw input must be transformed into useful input features, which is called the feature-extraction process. The features are obtained from genome shotgun sequences by dividing sequences into sub-sequences of length k, famously called k-mers [22,33,34,35,36,37], then by marking the frequency of presence or absence of each k-mer. Then, k-mers within a given sample can be counted and transformed into vectors, making 4^k^ possibilities. Typically, the length of a k-mer ranges between 13 and 31 nucleotides. Longer k-mers are more specific but are error-prone in sequencing and require more training data [22,34,35,36,53]. Some other techniques of feature mapping including mapping antimicrobial resistance genes, or pangenomes. By doing so, variations in novel genes and sequences can be obtained, and can capture features depending on the absence or presence of genes and/or SNVs [43,54,55,56,57]. Furthermore, meteorological/environmental data have been used to predict the percentage of the occurrence of different AMR environments such as water [28]. Such cases are useful in terms of informing under what season or condition there is more probability of infection spread [28].

### 2.4. ML/DL Models

The basic idea of the DL/ML model is to build a model using a huge amount of data to capture the underlying non-linear relationship between the input features and outcomes, which would be difficult otherwise [29,40,58]. All DL and ML models are trained first on the training dataset. Once trained, these trained models are ready to be tested with unseen data. The first step is to preprocess the data and extract important and relevant input features. Then, data must be split into training, test, and validation data. First, the model is trained by feeding the training dataset features. The training process will optimize and obtain optimal parameters. During training, certain parts of training is used to validate and improve the optimization. Cross-validation is also used to make the model robust [40]. A deep-learning model consists of different hyperparameters. For a given problem, an optimal combination of these hyperparameters produce optimal results. Therefore, different techniques of hyperparameter optimization, such as Bayesian optimization, should be used to obtain the optimal combination hyperparameters [29,40]. By optimizing the models and selecting appropriate parameters, accuracy can be much improved.

Different models are suited to different types of datasets, and accordingly high accuracy can be achieved by selecting appropriate models depending on the nature of the problem or objective. For instance, simple neural networks and recurrent neural networks (RNN) [29,40] are suitable for regression problems, while convolutional neural networks (CNN) [38] are suitable for classification problems. In the case of AMR, machine-learning and deep-learning models are both used for classification as well as regression. Similarly, decision-tree methods are good at classification, and these models are suitable for tracing back the performance [28]. 

Therefore, one of the most important parts of applying DL/ML is to select the most appropriate model depending on the application and type of input features. Complex models usually have high variance, while relatively simpler models have higher bias [59]. Simpler models are easy to interpret, but these models might show low accuracy when it comes to complex features. Therefore, it is important to select models carefully, considering their appropriateness in terms of identification and interpretation. For instance, the authors in [38,60,61,62,63,64,65,66] used deep-learning and machine-learning models to identify different antibiotics. The authors in [38] used traditional machine learning and CNN to rapidly predict tuberculosis drug resistance accurately from genome sequences. For instance, in [60], mutations relevant to antimicrobial resistance in *Mycobacterium tuberculosis* are highlighted by a convolutional neural network. Interesting antibiotics were discovered by the authors in [61] using machine-learning models. In [62], deep learning is used to identify antimicrobial peptides from the human gut microbiome. The authors in [63] identified the mechanism action of antibiotics using interpretable machine-learning models. The authors in [64] used a machine-learning pipeline, mining the entire space of the peptide sequence to identify potential antimicrobial peptides. The authors in [65] used deep-transfer learning to obtain the robust prediction of antimicrobial resistance for novel antibiotics.

It is also very important that these models should be easily and powerfully interpretable when it comes to the application in the health sector or diagnostics [67,68,69,70]. Interpretable models should be able to evaluate individual input features, be traced back and forth, and make it possible to analyze and use the interactions of multiple features that are impacting on the target [28]. Decision tree-based models are classifiers that work in a hierarchy of internal nodes to evaluate the features based on variance. These models apply an explicit decision criterion until the final stage is achieved by classifying into a particular group [67,71]. Therefore, each node of these models is traceable. Therefore, it is possible to understand the features or interaction of features responsible for a particular decision. Gradient-boosting models—ensemble methods from decision tree—have been successfully used in AMR prediction [28,72,73]. Table 1 shows different DL/ML models used in AMR identification and applications. The table classifies different techniques based on their merits and demerits when applied to AMR problems. 

### 2.5. Model Evaluation

**Figure 2:**
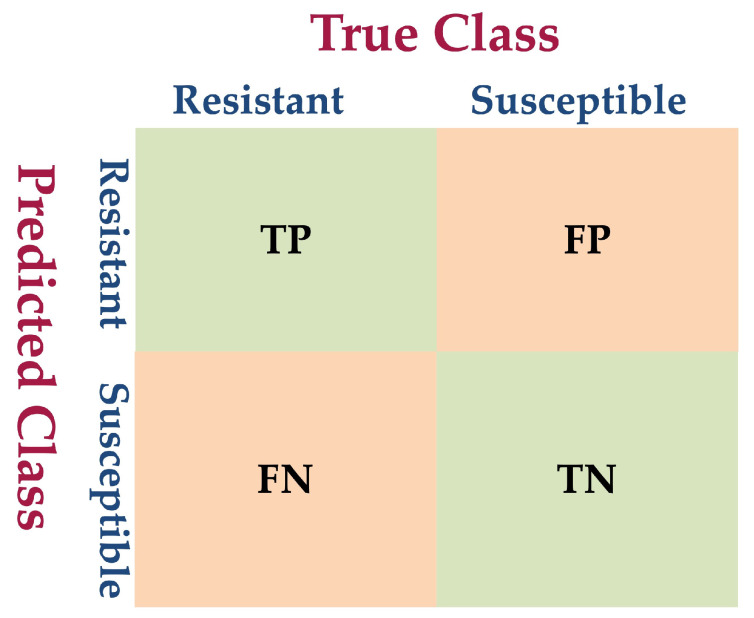
Confusion matrix for binary output classification problem.

Once the models are trained, they should be tested on different evaluation criteria. Different evaluation techniques indicate the strength of a model from different perspectives [41]. Usually, classification problems can be evaluated using a confusion matrix, more commonly when there are two class problems [41]. A confusion matrix evaluates the model based on the actual positive and negative cases against the predicted outcomes, which are true positives (TP), true negatives (TN) and false positives (FP) and false negatives (FN). TP stands for the actual positive that is predicted to be positive, and FP means actual negative cases that were predicted positives. Similarly, TN and FN can be explained. Figure 2 is an illustration of a confusion matrix of binary classification. Other evaluation criteria for classification are accuracy, recall and sensitivity [41]. In the case of regression problems, models are also evaluated based on accuracies and loss defined by root mean square error and correlation function, i.e., R2 score etc. [40] as given by equations in Table 2. This table gives the formula of different evaluation techniques.

### 2.6. Robustness of Different ML/DL Models in AMR Prediction

Table 3 shows the statical comparison of different models in terms of accuracy and robustness. For instance, the authors in [31] predicted resistance against antibiotics such as CIP, CTX, CTZ and GEN. The input data were obtained from *E. coli* strains which included around 1000 isotopes. SNPs were used as inputs. The SNPs were encoded in three different ways: label encoding, one-shot encoding, and CGR encoding. Four different models were trained, which includes CNN, LR, RF and SVM. Each model is separately trained for each type of encoding. On average, random forest regression predicted best for label encoding and CGR encoding with accuracies of 0.832 and 0.835, respectively. On the other hand, for one-shot encoding, CNN produced the best accuracy of 0.855. However, this research only considered SNPs, without considering longer genome sequences or k-mers. Therefore, it is difficult to find insights for the activities of components of the genome against these antibiotics. The authors in [66] also used the same data, but the objective was to identify new genes based on a deep CNN model. The idea was to train the base model against CIP. In addition, they used the trained weights of the CNN layers to predict the CTX, CTZ, and GEN with respective transferred models. However, the transferred model produced very low accuracies of around 40%. Therefore, a lot of improvement in these models is required.

The authors in [32] predicted AMR of *Actinobacillus pleuropneumonia* from WGS against five antibiotics (Tetracycline, Ampicillin, Sulfisoxazole, Trimethoprim, and Enrofloxacin). There were 96 isolated strains from *A. pleuropneumonia*. K-mers of the strain and the reference genes of the specific antimicrobials were used as input features. Two models were used, namely referenced-based SVM and reference-free Set Covering Machine (SCM). The accuracies of both the models were shown to be around 100%. This result indicates that the data were easily distinguishable between susceptible and resistance and did not include overlapping or intermediate cases. Therefore, such methods must be further investigated on different types of balanced data from different geographical locations. Otherwise, it would be difficult to use for practical purposes.

A strong deep-learning model, DeepARG [39] is compared with hierarchical multitask (HMD)-ARG by the authors in [30], to predict and classify ARGs. Input amino acids were encoded using one-shot encoding. The aim of the paper was to classify ARGs from non-ARGs, ARG antibiotic class classification, antibiotic mechanism classification, antibiotic mobility classification and beta-lactamese Amble classification. In ARG/non-ARG classification, the accuracy of DeepARG was 0.965 whereas that of HMD-ARG was 0.948. HMD-ARG was applied to classify the mechanism of ARG antibiotics with 0.936 accuracy. Furthermore, HMD-ARG was able to classify ARG antibiotic mobility with 0.909 accuracy. It also classified beta-lactamase Amble with 0.995 accuracy. However, in HMD-ARG, the inputs are assembled sequences, and its application scenarios may be limited, so cannot work on short reads unless heavy computational pre-processing is done. DeepARG [39] was trained on 30 categories of ARGs with the intention of predicting among these categories only; therefore, any unknown category/genes might not be predicted accurately. The objective of some other models and their limitations are shown in Table 3 as well.

## 3. ML/DL for AMR Prediction: Challenges and towards Practical Implementation

### 3.1. Challenges

Although artificial intelligence (AI) techniques can do wonders in finding AMR, paving new paths to rapid diagnostics, more accurate treatment, and cure, these opportunities come with challenges [6,20]. The mechanism of antibiotics and drugs is not completely understood, particularly in the case of newly arising diseases [6]. Furthermore, with mutations and other changes, it makes it more difficult to understand these mechanisms [77]. Additionally, the resistance behavior changes from cell level to micro-organism community level. For instance, in response to some cellular stressors [78] or antibiotics, a sub-population of bacteria might persist, and the capacity of a genome for resistance might quickly be augmented via HGT during biofilm formation [79]. These types of challenges are difficult to tackle, even if genome sequences are available. Therefore, AI models might struggle to learn the underlying mechanism with this type of evolution in resistance. However, deep-learning models, if provided with enough data and designed in a right way, would provide interesting findings [30,39,66].

Another challenge is that currently most AI models treat genes or sequences of genes separately, i.e., univariate. Although these models are accurate in prediction, the phenotypes are sometimes an outcome of a combination of genes or a combination of input features, producing a combined impact non-linearly [80]. For example, sometimes a combination of metals and antibiotic resistance genes combined produces certain AMR [80]. The maintenance and spread of AMR is suggested to be increased by association [81]. Although metal resistance might not directly impact antibiotics, its combination with AMR genes has shown enhanced resilience to AMR. Since most current AI models use single independent features, it is difficult to capture these types of collaborative or associative impacts. Minimal studies have been carried out to investigate the combined impacts of features or genes [82]. Too many multivariate features make it difficult and challenging to design models that can analyze and understand the interactions of impactful multivariate features to produce a certain outcome.

Another major concern is that, until recently, the general categorization of AMR classification was a binary classification of susceptible or resistant. Although ML/DL models show good accuracies in diagnosing highly resistant or susceptible genes [83], they might produce low accuracies if the intermediate category is also included. Designing models to add another category of an intermediate phenotype will make the overall outcome more effective in practical implementation. However, considering the intermediate category might have certain challenges. There is no standardized clearly defined boundary in a widely applicable documentation of antimicrobials between susceptible, intermediate, and resistant cases [84]. Furthermore, the definition of susceptible and resistant also keeps changing. This inconsistency of definition is summarized in [85]. Furthermore, intermediate isolates are far rarer, which might make the training and testing dataset imbalanced, causing incorrect assumptions or outcomes by the model. Complexity might arise by making multi-class classification in terms of interpretation and accuracies.

Overall limitations and challenges are summarized in this paragraph. The availability of data is a major concern. There are limited data available on AMR, particularly for less common micro-organisms or for those that have been isolated from unusual environments [49]. This can make it difficult to train effective machine-learning models. Furthermore, the quality of data is another challenge. The quality of the data used to train machine-learning models can have a significant impact on performance. Poor-quality data, such as data that is noisy or contaminated, can lead to inaccurate results. Another challenge is the imbalance of data. In some cases, the data used to train machine-learning models may be imbalanced, with a disproportionate number of samples belonging to one class (e.g., resistant or susceptible) and not including the intermediate class [84]. This can make it difficult for the model to accurately classify the minority class. Identifying the most relevant features for use in machine-learning models is challenging too. It is important to select features that are predictive of AMR, but also to avoid including redundant or irrelevant features, as this can negatively impact model performance. Machine-learning models can be prone to overfitting, especially when they are highly complex [66]. This means that they may perform well on the training data, but poorly on unseen data. It is important to carefully tune the complexity of the model to achieve good generalization performance.

### 3.2. Towards Practical Application of AI in the Antimicrobial Sector

**Figure 3:**
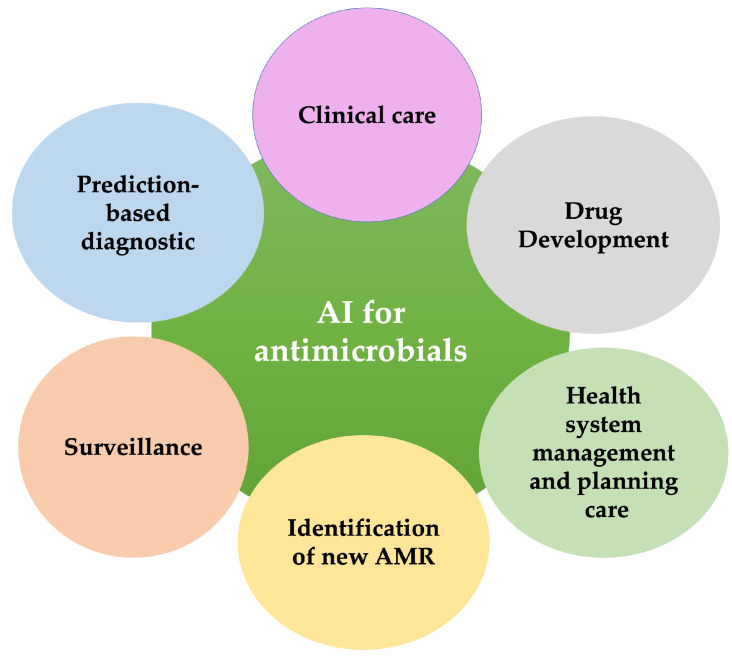
AI can be applied on antimicrobials to obtain different objectives such as clinical care, drug development, surveillance, identification of new AMR etc.

Currently, most of the research is restricted to laboratories and not yet implemented practically, although many research works are underway to make them practically applicable. Figure 3 summarizes prospective AI application in the AMR sector. Models are developed for hypothesis deduction on new AMR genes or mutation-variation mechanisms [86]. The outcomes of prediction from some models have been tried to be applied in diagnostics [51]. Integrating genomics to improve surveillance is becoming a hot topic of discussion [87]. Emerging AMR trends can be shown by monitoring known causal resistance genes, and transmission patterns can be revealed that can help in identifying and controlling outbreaks of resistant pathogens. Whole-genome sequence data are expanding, helping AI models to obtain high accuracies in surveillance [88,89,90]. AI models can learn highly impactful features, so that necessary steps can be taken beforehand.

Traditional practices of antimicrobial diagnostics are neither rapid nor intuitive. For instance, more than 24 h is required to perform the current susceptibility test, and it requires the expertise and care of a bioinformatician with minimal error to perform the whole-genome sequence of an antibiotic susceptibility test and huge amount of data is necessary for it to be processed [91]. Different studies have been carried out to minimize the time of diagnosis. For example, diagnosis time can be reduced to 3 h using a flowcytometry antimicrobial susceptibility test and ML [23]. An efficient way of genome data management using artificial intelligence is given in [92]. The establishment of optimal antibiotic use strategies in sepsis treatment using AI-based data-driven techniques is presented in [93]. Specifically, artificial intelligence can positively identify suitable actions, predict with high accuracy the mortality and the length of stay, improving the patient outcome. Commonly used phenotype test methods could have an accuracy of around 90% with Phoenix [94]. ML/DL models can improve these accuracies, if provided with appropriate data and training mechanisms. A revolutionary antimicrobial stewardship would be to design a tool that used personalized medicine based on quickly detecting a pathogen and its resistance profile from clinical samples. For example, in sepsis, mortality is adversely impacted by up to 20% by delayed effective antibiotic therapy [95]. Interests are also increasing in fast diagnostics for bloodstream infection. Such steps, in combination with antibiotic stewardship, would increase the outcome of patients [96]. Different techniques of artificial intelligence are applied in silico to predict new antibiotic molecules and to investigate synergy from drug combination [97]. Since 2014, around 14 new antibiotics have been developed and approved, and the application of artificial intelligence can speed up the pace of antibiotic discovery and production [98].

Artificial intelligence is also playing its part in ensuring clean water supply and good hygiene. Different ML models can predict the chances of the occurrence of antimicrobials in water [28]. Another work has been performed using AI to harness the water crisis [99]. The idea of this work is to ensure access to clean water and sanitation by reducing infectious diseases and the spread of AMR bacteria. Some major practical applications are water resource management, contamination detection, effluent quality improvement, and the monitoring of data.

**Figure 4:**
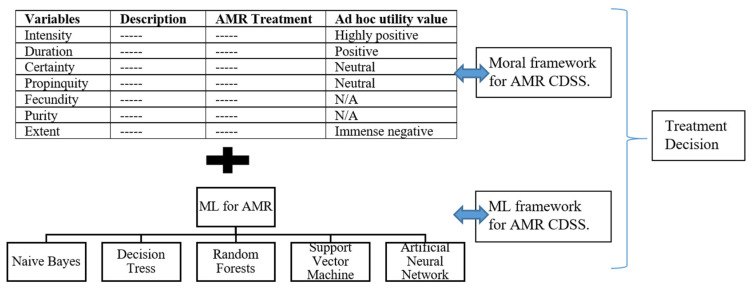
Combination of moral and AI-based frameworks for CDSS.

**Figure 5:**
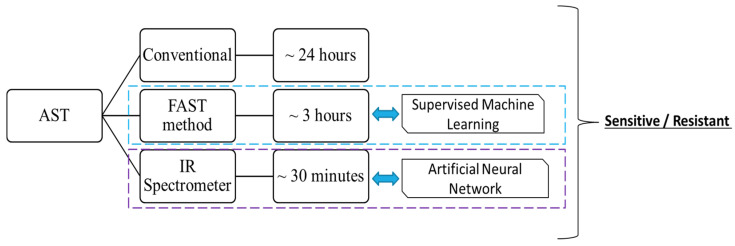
Efficiency of AST methods based on AI and conventional techniques.

For illustration, some practical examples are given in Figure 4 and Figure 5. Figure 4 illustrates a framework known as Bentham’s felicific calculus and its application to the decision to start antimicrobial treatment. It is an AI-based clinical decision support system (CDSSs) for antimicrobial optimization considering a moral framework known as Bentham’s felicific calculus. This framework helps to start treatment based on the ML outcome and moral framework [100,101]. Figure 5 shows antimicrobial susceptibility testing (AST), which is one of the most widely used methods for the diagnoses of AMR [102]. The conventional methods are not efficient, need huge datasets, and take more time compared to the AI-based techniques [51,103,104]. Studies have applied supervised machine learning to improve the AST method and reduced the conventional 24 h to a mere 3 h for the test [23,105]. Similarly, Lechowicz et al. developed an artificial neural network-based IR-spectrometer test to reduce AST time to just 30 min [24,25].

## 4. Conclusions

This article has reviewed state-of-the-art artificial intelligence in tackling antimicrobial-related challenges and opportunities. Artificial intelligence is doing wonders in different domains of humanity. Deep learning and machine learning are subfields of artificial intelligence, tackling challenges by using huge amounts of data. Presently, huge amounts of data related to antimicrobials can be obtained from different sources. Additionally, very powerful processing computers with the help of large storage devices can process these data in almost no time, giving interesting insights. This has led researchers in the antibiotic sector to use AI tools to solve challenges. Currently, a lot of work on the application of AI in antibiotics is under progress, which has opened new pathways. For instance, the amount of time taking in diagnostics is highly reduced from days to hours by applying AI. Furthermore, new AMR and mutations are being discovered with the help of AI. Antimicrobial quantity can be predicted in water resources, and so on. However, there are certain major challenges in the application of AI on AMR. For example, most applications consider only the output to be resistant or susceptible, without considering an intermediate category that overlaps the susceptible and resistant categories. This can produce a wrong diagnosis. Furthermore, generally univariate features are analyzed and related to antibiotic genes. However, it is known that multiple features are responsible for generating or identifying AMR. Therefore, multivariate/interactive models need to be designed. Results obtained by training imbalanced data are not dependable. Models are mostly trained on sequences of a particular geography that might not produce universal output. Data management is another big concern. The research on the application of AI into AMR is still underway, and more is yet to be discovered before consideration for clinical and healthcare application on an extensive level.

## Figures and Tables

**Table 1:** Different ML/DL models and their merits and demerits in AMR problem applications.

Technique	Algorithm	Advantages	Disadvantages
Neural Networks (simple Neural networks, RNN, CNN etc.) [38,60,61,62,63,64,65,66]	These models mimic the human brain and learn by optimizing weights until the final objective is achieved. The better the data, the better is performance Can perform on multi-dimensional data	✓ Can solve complex problems ✓ Feature interaction ✓ Can evaluate features ✓ Capable of multivariate features	✕ Increased model complexity with increase in layers and nodes ✕ The model is not traceable
Decision Tree [28,72,73]	Predict based on target. Leaf nodes equal class label, nodes in the model equals to attributes	✓ Can evaluate features ✓ Model is traceable ✓ Feature interaction	✕ Model might suffer with increasing feature complexity/multivariate
Logistic Regression [74]	Logistic curve that associates to each input features	✓ Feature evaluation	✕ Non-traceable ✕ Interaction of features is not possible

**Table 2:** Evaluation metrics used in different ML/DL problems.

**Regression/Prediction**	
**Evaluation Matrix**	**Formula**
Root Mean Square Error	$\sqrt{\frac{1}{N}.\sum_{n=1}^{N}{\left(y_{n}^{\prime }-y_{n}\right)}^{2}}$
R2 score	$1-\frac{\sum_{n=1}^{N}{\left({y^{\prime }}_{n}-y_{n}\right)}^{2}}{\sum_{n=1}^{N}{\left(y_{n}-\overset{-}{y}\right)}^{2}}$
**Classification/Prediction**	
Accuracy	$\frac{TP+TN}{TP+FP+TN+FN}$
Recall/Sensitivity	$\frac{TP}{TP+FP}$

$N\;\mathrm{is}\;\mathrm{total}\;\mathrm{samples},\;y_{n}^{\prime }\;\mathrm{is}\;\mathrm{predicted}\;\mathrm{values},\;y_{n}\;\mathrm{is}\;\mathrm{the}\;\mathrm{actual}\;\mathrm{values}\;\mathrm{and}\;\bar{y}\;\mathrm{is}\;\mathrm{the}\;\mathrm{same}\;\mathrm{value}.$

**Table 3:** Comparison of different ML/DL models for AMR prediction.

Objective	Features	Models	Model for Comparison	Performance	Remarks
Predict AMR (such as CIP, CTX, CTZ and GEN.) [31]	SNPs are being encoded	CNN, RF	LR and SVM	With label encoding RF showed 0.83, with hot encoding, CNN showed 0.855, and with CGR encoding RF showed 0.835	Only SNP data used called based on a single reference genome
Evaluate Machine-Learning models to Predict AMR [32]	k-mers of the strains from WGS	Referenced SVM, and Reference-less SCM		Both models produced around 1.00 precision	Very high precision indicates data are not well balanced
Deep-Transfer Learning to predict Novel AMRs [66]	k-mers and SNPs being encoded	Deep CNN-based transfer leaning		Basic model produced 0.83, transferred models for novel resistance produced less than 0.41	Transferred models producing less precision
Annotating antibiotic resistance genes [30,39]	Genome represented by k-mers	HMD-ARG	Deep-ARG	ARG/non-ARG classification accuracy of 0.948 and antibiotic mobility 0.909	Inputs are assembled sequences, its application scenarios may be limited, and cannot work on short reads unless heavy computational pre-processing are done
Predict vancomycin intermediate susceptible *S. aureus* phenotype [75]	Resistance genes identified in past	LR	Multylayer perceptron, SVM and RF	Correctly classified 21 out of 25	Model is being built using only 25 genomes
Predict carbapenem resistance in *A.* *baumanii*, methicillin resistance in *S. aureus*, and beta-lactam and co-trimoxazole resistance in *S. pneumoniae* [76]	Bacterial genome represented by k-mers	AdaBoost		A *A. baumanii*, *S. aureus*, *S. pneumoniae*: 88–99%. *M. tuberculosis*: 71–88%.	No comparison algorithms used. Approach now implemented as classification tool on Pathosystems Resource Integration Center website

## Data Availability

Not applicable.
